# Association between Microorganisms and Microplastics: How Does It Change the Host–Pathogen Interaction and Subsequent Immune Response?

**DOI:** 10.3390/ijms24044065

**Published:** 2023-02-17

**Authors:** Wenjie Yang, Yang Li, Diana Boraschi

**Affiliations:** 1 Shenzhen Institute of Advanced Technology (SIAT), Chinese Academy of Sciences (CAS), Shenzhen 518071, China; 2 China-Italy Joint Laboratory of Pharmacobiotechnology for Medical Immunomodulation, Shenzhen 518055, China; 3 Institute of Biochemistry and Cell Biology, National Research Council, 80131 Naples, Italy; 4 Stazione Zoologica Anton Dohrn, 80132 Naples, Italy

**Keywords:** microplastics, innate immunity, bacteria, viruses, host–pathogen interaction

## Abstract

Plastic pollution is a significant problem worldwide because of the risks it poses to the equilibrium and health of the environment as well as to human beings. Discarded plastic released into the environment can degrade into microplastics (MPs) due to various factors, such as sunlight, seawater flow, and temperature. MP surfaces can act as solid scaffolds for microorganisms, viruses, and various biomolecules (such as LPS, allergens, and antibiotics), depending on the MP characteristics of size/surface area, chemical composition, and surface charge. The immune system has efficient recognition and elimination mechanisms for pathogens, foreign agents, and anomalous molecules, including pattern recognition receptors and phagocytosis. However, associations with MPs can modify the physical, structural, and functional characteristics of microbes and biomolecules, thereby changing their interactions with the host immune system (in particular with innate immune cells) and, most likely, the features of the subsequent innate/inflammatory response. Thus, exploring differences in the immune response to microbial agents that have been modified by interactions with MPs is meaningful in terms of identifying new possible risks to human health posed by anomalous stimulation of immune reactivities.

## 1. Introduction

The widespread and substantial accumulation of discarded plastic products has changed the environment. The majority (almost 80%) of produced plastic is released into the environment, mainly through burial, direct disposal, and dumping in water bodies [1]. Discarded plastic can be found in the air, food, soil, and marine and freshwater environments, as well as in organisms living in these environments [2,3,4]. It has been estimated that more than 800 million tons of plastic enters the marine environment each year, and it can be expected that similar amounts are present in landfills and freshwater [5]. Microplastics (MPs) are plastic fragments smaller than 5 mm derived from the degradation of plastic products in natural environments (e.g., water and soil). MP intake by children and adults was calculated to be more than 500 and 880 particles per day, respectively [6]. MPs have an elevated ratio of surface area to volume, which enhances their ability to adsorb other substances [7,8]. Furthermore, the physical–chemical properties of MPs can substantially change with the weathering process (e.g., irradiation, erosion, etc.) [9,10], a process that can amplify and diversify the capacity of MPs to adsorb and transport different chemical contaminants [11,12] and biological agents present in the environment (e.g., viruses, bacteria, and allergens). Thus, microbial agents can be taken up by living organisms through ingestion or inhalation of MPs and even by dermal contact [2,10,13,14,15,16,17]. We expect that the biotic–abiotic association between MPs and microorganisms may change their interaction with the host barrier and defense mechanisms, including immune responses, in both environmental species and human beings. In this context, we will examine how associations with MPs can, on one hand, affect the intrinsic biological properties of microorganisms (e.g., bacteria, viruses, and their components) and, on the other hand, change the features of the interactions between microorganisms and cells of the immune system, thereby facilitating or hampering recognition and defensive reactivity.

## 2. Interactions between Microorganisms and Microplastics

Microorganisms are known to colonize MP surfaces. Interactions between microbes and MPs depend on the MP’s surface characteristics (including size, shape, roughness, and hydrophobicity), as well as environmental factors, such as temperature and the microenvironmental pH and ionic strength, since interactions with external agents are mainly driven by hydrophobic and electrostatic forces [18]. In the case of bacteria, the initial interaction occurs through electrostatic forces and depends on the MP’s size, chemical composition, and surface modifications [19]. MPs can provide a suitable substrate growth area for microbial communities and important nutrients for their growth (which are adsorbed on their surface from the environment, e.g., metal ions such as zinc, iron, and copper). Bacterial growth is facilitated by the rough surface of weathered MPs [20], with the generation of biofilms, as has been shown for *Vibrio* species [21,22]. Many microorganisms have been found on the surface of MPs, including bacteria such as *Aeromonas*, *Rhodococcus*, *Pseudomonas*, *Enterobacter*, *Halomonas*, *Mycobacterium*, *Photobacterium*, and *Shigella*, and fungi [23]. Notably, the bacterial communities on MPs (the “plastisphere”) are significantly different from those in the surrounding seawater [21,24] and are also different depending on the MP’s composition and characteristics (e.g., polyethylene vs. polypropylene, biodegradable vs. non-degradable, and different degrees of hydrophobicity), indicating colonization selectivity [25]. The toxicity of MPs for both autotrophic and heterotrophic bacterial growth has been observed and is mainly due to toxic organic additives leaking from the MPs [26]. 

Substantial evidence suggests that viruses can attach to plastic surfaces [27]. For example, SARS-CoV-2 can attach to polypropylene surfaces and survive for more than 72 h, which is longer than on copper or cardboard [28]. Viruses can bind to naked polypropylene plastic surfaces through non-ionic forces [29]. In a recent study, SARS-CoV-2 showed elevated infectivity if attached to polystyrene MPs because of preferential uptake and shuttling to endo-lysosomes, whose low pH facilitates viral replication [30]. The study showed that the virus did not change when it interacted with MPs, but adsorption on MPs changed the pathway of viral entry into cells (i.e., taken up by phagocytosis and shuttled to endo-lysosomes rather than fusing to the plasma membrane and reaching the cytoplasm). In other situations, adhesion to plastic surfaces can inactivate viruses, as in the case of *Poliovirus 1* when it is stored in a hydrophobic polypropylene container in groundwater [31]. Thus, the fate of viruses interacting with MPs seems to depend on the type of plastic and the type of virus. The notion that some viruses can survive on MP surfaces for long periods of time supports the hypothesis that viruses can hijack particles for transportation and spreading. Co-existence of bacterial colonies and viruses on MPs is likely, although it has never been proven experimentally.

Even in the absence of living microorganisms, microbial components released from dead bacteria can bind to MP surfaces [32]. Since many bacterial components can trigger sterile inflammation by interacting with Toll-like receptors (TLRs) on the surface of immune cells, adhesion of lipopolysaccharides (LPS) or other bacterial TLR agonists to MPs may expand the inflammatory effects of such TLR agonists both by concentrating them and by broadening their range of action. The ability of bacteria to form biofilms on MP surfaces promotes the interaction of MPs with the many bacterial-derived molecules that form extracellular polymeric substances (EPSs), which mainly consist of polysaccharides, extracellular DNA, and proteins [33]. Among them are DNABII proteins [34] and other histone-like proteins (HU), which promote the attachment and growth of other bacterial species when released into the EPS after microorganism death [35]. HU can also act as a “molecular glue” for binding LPS [36]. It is possible that adhesion to MPs may change the structure of microbial biomolecules, although there is no evidence that this can actually happen. A simulation study compared four different nano- and micro-sized MPs (PE, PP, PET, and nylon) and predicted that there would be alterations in protein secondary structures (α-helix and β-sheet) [37], but no structural changes of real proteins were ever observed.

Other microbial molecules that can adsorb on MP surfaces are antibiotics, such as tetracyclines, macrolides, fluoroquinolones, chloramphenicol, and sulfonamides, which can bind by hydrophobic and electrostatic forces. The presence of antibiotics can promote the development of antibiotic-resistant genes (ARGs) in surrounding bacteria and in extrachromosomal DNA, which can easily be transferred between bacterial communities and different species [38,39]. A recent study demonstrated that mice ingesting MPs and sulfamethoxazole had less tissue accumulation of the antibiotic but a significantly elevated ARG profile in their gut microbiota compared to ingestion of the antibiotic alone, suggesting a change in the pharmacokinetics and pharmacodynamics of the antibiotic due to its interaction with MPs [40]. 

Among other biological molecules in the environment, it is likely that MPs can act as carriers for antigens and allergens and, upon inhalation or ingestion, induce an immune reaction that differs from the reaction induced by isolated antigens/allergens. Moreover, it has been established that exposure to MPs can induce an inflammatory reaction and barrier impairment in the digestive system and lungs, thereby exacerbating pathological reactions to food and inhaled allergens [41,42]. In the case of dermal contact, exposure may occur through contaminated soil, settled dust, textiles, and cosmetics [16,17,43,44,45,46]. MPs can cross the skin barrier either directly, if smaller than 100 nm [14], or through hair follicles and sweat glands [47]. In addition, MPs can penetrate the skin through open wounds and even provoke skin damage by inducing an inflammatory reaction [13,47], thereby allowing the entry of associated microorganisms.

**Figure 1:**
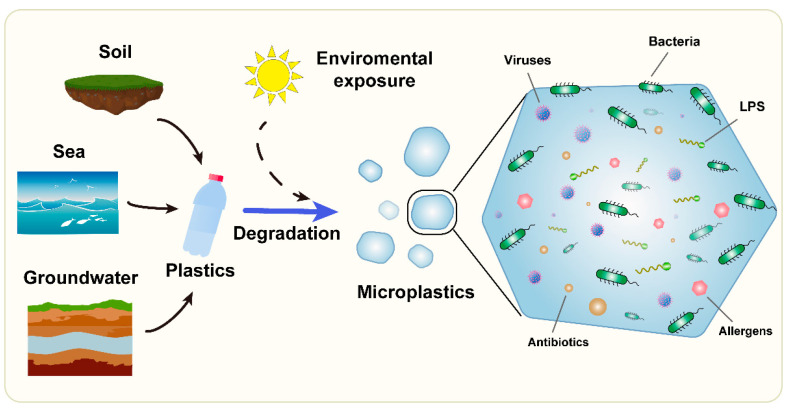
Microplastics as carriers of microorganisms and related molecules.

A representation of the interaction of microorganisms and related molecules with MPs is shown in Figure 1.

## 3. Immune Reactions to Microorganisms Complexed with Microplastics

Innate immunity at the tissue barrier is the first immune defense mechanism to restrict and neutralize external threats, especially microorganisms and foreign particles that have overcome the mechanical and chemical barriers. In human beings, such barriers include surfactant and mucus (in the lungs and digestive system), stratum corneum (in the skin), flux generated by coordinated ciliary activity (in the respiratory tract), peristaltic movement (in the digestive system), low pH (in the stomach), and epithelial/mucosal layers. The vast majority of particles and microorganisms do not overcome these barriers, although MPs have been reported to cause alterations (for instance, by selective adsorption of some surfactant components) that can hamper organ functional integrity [48,49]. The entry and accumulation of MPs in organs generally depends on their concentration, size, and shape [14,15,50,51,52,53]. Size plays a substantial role in particle uptake. For example, compared with 20 μm MPs, 5 μm MPs can accumulate more abundantly in mouse intestines and kidneys [54]. Polystyrene (PS) MPs of 1 μm do not enter human or mouse hepatocytes, while 0.1 μm PS MPs are able to do so [53]. Likewise, cells of the human colon cancer cell line Caco-2 were shown to uptake more than 50% of PS MPs smaller than 1 μm in vitro, while larger particles were hardly taken up at all [55]. Thus, a limited number of inhaled/ingested particles actually come in contact with cells and factors of the innate immune system; they are most likely on the nanoscale. A similar situation may also occur in invertebrates. For example, in filter feeder marine invertebrates, the majority of large particles were expelled from the gut without having the opportunity to accumulate in tissues [56]. Smaller MPs have a larger surface area compared to larger particles, which may favor the carryover of toxic molecules (such as bisphenol A and BPA) [55] and, most likely, any other adhered molecules or microorganisms. Thus, the passage of microorganisms attached to small MPs may be facilitated, allowing them to overcome barriers and invade the underlying tissue. After overcoming the mechanical barriers, both types of foreign agents (MPs and microorganisms) can be sensed and tackled by innate immune mechanisms present in the affected tissue, particularly soluble factors, such as complement and antimicrobial peptides, and effector cells, such as macrophages, mast cells, and innate lymphoid cells [57]. If foreign agents induce an inflammatory reaction, then other innate effector cells, particularly neutrophils and monocytes, will be recruited from the blood.

An important outstanding issue is whether adhesion to MPs may change the capacity of the immune system to recognize and eliminate pathogens. As discussed earlier, adhesion to MPs may select the type of microorganism, promote biofilm formation, and concentrate specific strains. A recent study showed that *Helicobacter pylori* could form a biofilm on the surface of polyethylene MPs, and the bacteria/MP complex accelerated and exacerbated *H. pylori*-induced gastric injury and inflammatory response in a mouse model [58]. While the study cannot discriminate between mechanical MP-dependent injury, as the cause of the increased infectivity, and MP-dependent increased infectivity, as the cause of gastric injury, it is clear that the combination changed the host’s interaction with the pathogen and increased pathogenicity. In the same study, ingested MPs were found in the liver, suggesting the possibility that they were translocated to the inner organs, although they could not exclude the possibility that damage to the stomach mucosa promoted passive entry into the inner tissues.

Thus, microorganisms may hijack MPs, and use them to enter the body in non-canonical ways. In this way, microorganisms can overcome immune defense mechanisms that developed over the course of evolution to tackle them through canonical pathways. Conversely, recognition of microorganisms adhering to MPs, i.e., a complex larger than the microorganism itself, may shuttle them more easily toward destruction by phagocytes. A very interesting example is that of SARS-CoV-2, which can infect cells in two main ways [59]. First, virus interaction with the ACE2 receptor on target cells can occur at the level of the plasma membrane, leading to fusion of the virus particles with the membrane and release of viral RNA into the cell cytoplasm. The second mechanism leads to more efficient infection and encompasses the uptake of viral particles within endosomes: interaction with ACE2 on the luminal endosomal membrane, acidification of the endosomal compartment, fusion to the endosomal membrane, and release of uncoated RNA in the cytoplasm. It has been shown that adhesion to MPs preferentially shuttles the virus toward the second mechanism, thereby increasing viral infectivity by favoring the more efficient infection pathway [30]. However, when the virus adhered to an MP, it could be taken up with high efficiency by macrophages [60], which are otherwise unable to do so as they do not constitutively express the SARS-CoV-2 receptor ACE2 [61,62,63]. Once within the phago-lysosomes, viral fusion to the vesicle membrane cannot take place because of the absence of ACE2, and the virus is efficiently degraded by lysosomal enzymes [64]. Thus, adhesion to MPs can, on one hand, increase infectivity in ACE2-bearing cells and, on the other hand, facilitate virus destruction by ACE2-negative phagocytes.

Although very small particles can enter cells through passive transport, the majority are taken up by active transport endocytic/phagocytic mechanisms [65]. Macrophages are specialized phagocytic cells whose main role is to take up and destroy endogenous damaged matter and foreign agents, thereby eliminating possible threats to tissue integrity [66,67,68]. Macrophages can phagocytose particles up to 20 μm in size [69] and of different shapes [70]. Macrophages also recognize particle stiffness and take up stiff matter more easily than soft compressible particles [71]. If particles cannot be digested/degraded by lysosomal enzymes, macrophages simply sequester them in intracellular vesicles or eliminate them by exocytosis, with other macrophages taking them up [72]. The uptake of particles by macrophages is strongly affected by the substances present on the particle surface. In the case of MPs, the uptake of plasma-coated particles was shown to be significantly greater than that of non-plasma-coated MPs [73], and the amount of proteins in the particle “biocorona” correlated with their uptake by macrophages [74]. Likewise, MPs exposed to different environments (marine vs. freshwater) were taken up by mouse macrophage-like cells much more abundantly than naked particles, stressing the importance of the environmentally acquired biocorona in determining their interaction with phagocytes and particle uptake/elimination [60]. 

Besides phagocytosis, a very effective antimicrobial defense mechanism is the use of neutrophil extracellular traps (NETs), bundles of DNA-based filaments decorated with vesicles and enzymes released by neutrophils that are able to trap and degrade microorganisms extracellularly [75]. Neutrophils enter a tissue site only when an inflammatory reaction takes place, meaning that NETs usually form only during an active inflammatory event. Thus, NET formation is mainly an inflammatory event, largely consequent to cell death (NETs are usually released upon neutrophil death), and is therefore part of a non-homeostatic active defensive immune mechanism. Besides neutrophils, however, other cells, such as tissue macrophages and mast cells, can release extracellular traps, [76,77,78,79,80,81,82,83]. Thus, these extracellular traps appear to be an important innate defense mechanism, even in homeostatic conditions, able to entrap and eliminate particulate agents, complementary to phagocytosis. NETs can also entrap and degrade engineered nanoparticles, as shown for carbon nanotubes [67,75,84,85,86]. The finding that nanoscale PS MPs (of the size that can enter cells) induced NET formation in mouse neutrophils in vitro may indicate inflammatory activation and/or induction of cell death [87], as well as ROS production, which is another important element in NET formation [88]. How these observations may relate to NET induction in vivo in response to MPs that carry bacteria on their surface is still unknown, but it is likely that NET formation occurs in response to bacteria/MP complexes, in particular for complexes of micrometric size. Indeed, neutrophils can make appropriate adjustments to the size of exogenous agents so that NETs mainly capture large microorganisms, while small agents are mainly taken up into phagosomes [89]. 

**Figure 2:**
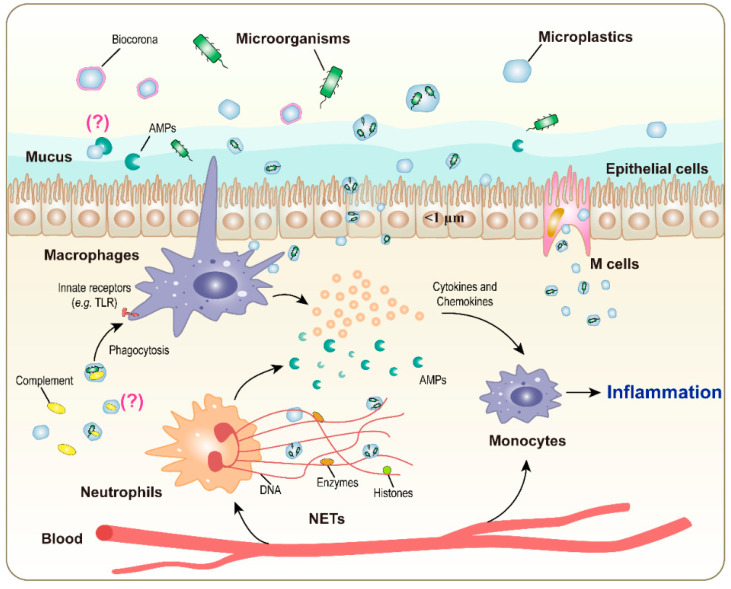
Ways of interaction of the immune system with microorganisms complexed with microplastics at a mucosal site.

Among soluble innate immune effector molecules, the proteins of the complement system are of particular interest. Complement proteins can recognize and bind ordered surface molecular patterns typical of bacteria and initiate a lytic cascade or, by coating the microorganism surface in a process called opsonization, promote phagocytosis by binding to complement receptors on phagocytes [57]. Complement activation by nanoparticles is a well-known phenomenon that can occur in the circulation despite the stealthing effect of the serum biocorona [90,91,92,93,94]. Whether complement components can directly adsorb on MP surfaces is not known (Figure 2). On the other hand, there are data suggesting that bacteria colonizing MP surfaces can be less susceptible to the complement attack. In particular, since adhesion to MPs can promote bacterial growth and the formation of biofilms, there will be a reduction in the capacity of complement to bind and opsonize bacteria, as well as promote their phagocytosis and lysis, as in the cases of *Streptococcus pneumoniae* [95], *Pseudomonas aeruginosa* [96], and *Staphylococcus epidermidis* [97]. In addition, the increase in the size of microorganisms carried on MPs, as well as the coating with opsonizing complement molecules that occurs in contact with biological systems, may trigger the elimination mechanisms of phagocytes (phagocytosis if the particles are not too large, NET formation and encapsulation if they are larger), which, together with the particles, will also eliminate the microorganisms on their surface [57]. Other important innate effectors are antimicrobial peptides (AMPs) (e.g., cathelicidins, defensins, and histatins), which are mainly produced by epithelial cells and phagocytes and are broadly active against bacteria, viruses, and fungi [98,99]. Cationic AMPs can easily adsorb on the surface of plastic or glass [100]. Whether such adsorption affects their capacity to kill bacteria or whether they can prevent bacterial colonization of MPs is currently unknown (Figure 2). 

Finally, it is important to assess how the adsorption of microorganisms by MPs can affect the induction of innate immune memory, which is a highly conserved protective mechanism present in most living organisms (plants, invertebrates, and vertebrates) [101,102]. Microorganisms and their components, such as LPS, can induce protective memory in macrophages and other innate cells, resulting in a decreased or enhanced response to subsequent challenges (optimized responses to tackle upcoming infections without excessive self-damage) [103,104]. While it has been found that engineered nanoparticles are able to directly induce memory [105] or modulate bacterially induced memory [106], no information is currently available regarding the possible effects of MPs on innate immune memory induced by bacteria or bacterial components adsorbed on their surface. 

A schematic representation of the known and putative interactions between MP/microbial complexes and the immune system is presented in Figure 2.

## 4. Conclusions and Perspectives

The notion that viruses, bacteria, and microbial components can adhere to MPs is bound to change our understanding of pathogen–host interactions. Microbes can use MPs (as well as other particles) to increase their size, change their characteristics, and exploit alternative interaction modes with immune cells. Most MPs in the environment (due to being subjected to wearing and aging) are spherical in shape, with a large surface area compared to size, and are therefore more prone to adsorb environmental factors. Microorganisms and their components are among these factors; therefore, the formation of MP/microbial complexes is highly likely. The most likely routes of human exposure to such complexes are skin contact, inhalation, and ingestion. Although barrier tissues have powerful ways of keeping particles and potentially harmful agents at bay, some of these complexes can nevertheless gain access to the inner tissue, where they come in contact with innate immune cells and factors. An outstanding question is how the immune system reacts to these complexes. An inflammatory reaction may occur, provoked by the size of the complexes and their chemical characteristics (pertaining to both the MPs and the microorganisms). The type of cells involved in these interactions is very important, with some interactions leading to enhanced elimination and clearance and others provoking pathological effects. Table 1 summarizes the main issues relative to the interactions between human immunity and microorganisms on MPs.

Thus, we propose the following questions and possible answers to be used for future directions.

1. Do MP/microorganism complexes cause more harm than microorganisms or MPs alone?

This cannot be stated with certainty. MPs have a size, shape, and chemical profile that changes when they interact with microorganisms. Likewise, the types of microorganisms that colonize MP surfaces are determined by the chemical features of the MPs. Depending on the MP characteristics, they can form biofilms, which makes them more resistant to recognition and elimination by immune defenses. However, the ways in which MP/microorganism complexes can overcome barrier tissues and gain access to inner tissues is not known. It is most likely that larger particles are eliminated/expelled more easily, implying that the complexes are less likely to enter the body than their isolated components.

2. Are MP/microorganism complexes eliminated more easily by phagocytes than isolated microorganisms?

The answer seems to be yes. Phagocytes can take up the complexes by phagocytosis (or endocytosis if smaller in size); furthermore, if the complex is very large, they can use NETs extracellularly. MPs are not significantly degraded by leukocyte enzymes, but microorganisms on their surfaces can be killed and degraded. Undigestible phagocytosed particles can be kept within tissue macrophages for a very long time [72], while particles that are too large to be phagocytosed can be surrounded and isolated from healthy tissue. However, changes in the interactions between microorganisms and immune sensing/reaction mechanisms can also facilitate infection. Some bacteria, such as *Mycobacteria*, can survive within macrophage phago-lysosomes and actually use these cells for spreading [107,108]. Many viruses infect cells upon fusion of their capsid with the plasma membrane of the target cells and subsequently release their nucleic acid into the cell cytoplasm. For this reason, cells are endowed with cytoplasmic virus-sensing systems that can detect the presence of viral molecules and initiate a defensive reaction (RIG-like receptors are one of these sensing systems) [57,109,110]. Adhesion of viruses to the surface of MP particles would substantially change the pathway viruses use to enter cells, which would mostly be by phagocytosis, thereby circumventing the cytoplasmic sensing/reaction systems. Since the mechanisms of immunity are always redundant (different systems in different locations within cells, different cells or soluble factors within tissues), it is therefore expected that other defense mechanisms would come into play if a microorganism enters a cell using an unconventional pathway. For example, bacterial components are not only sensed by receptors on the plasma membrane and, following phagocytosis, in the phagosome but also by sensors in the cytoplasm (NOD-like receptors), implying that both extracellular bacteria and bacteria/bacterial components that gain access to the cytoplasm can be recognized and trigger a defensive response [57]. This would suggest that, despite a different entry route, the wide range of innate immune mechanisms may be able to effectively deal with MP-associated microorganisms.

3. Is MP shape and size important in determining immune reactions to MP/microorganism complexes?

The answer is most likely yes, because the shape and size of MPs will dictate the shape and size of their complexes with microorganisms; consequently, this will determine the capacity of innate immune cells to detect them and the way in which the immune cells react (e.g., NET formation vs. phagocytosis). The shape of MPs that have formed over time during environmental degradation is most likely spherical, or at least smooth, but sharp edges may be present on MPs derived from rigid plastic materials, or fiber-shaped particles may be generated. In addition, MPs may contain toxic additives or have adsorbed toxic compounds. Thus, being of a large size, having a fiber-like shape or sharp edges, or containing toxic compounds may give an MP the capacity to provoke damage to the tissues with which it comes in contact. Since these are generally barrier tissues (e.g., skin and mucosae), the MP-caused damage will breach the barrier and allow associated microorganisms to enter. This scenario implies the development of a strong innate/inflammatory reaction aimed at restricting microbial invasion before re-establishing the barrier’s integrity. If the reaction is excessive or prolonged, it can cause severe tissue damage and become pathological. Another way in which MPs may alter immune defenses is when they are taken up into endo- or phago-lysosomes. If they have sharp edges, they can pierce the vesicle membrane and release into the cytoplasm not only the associated microorganisms and their components (which will be sensed by NOD-like receptors and other cytoplasmic innate systems) but also several proteolytic enzymes, which are able to damage cytoplasmic proteins and organelles, as well as trigger a strong inflammatory reaction (i.e., by activating the inflammasome), autophagy, and eventual cell death [111,112,113]. On the other hand, phago-lysosomal membrane destabilization may have advantages in terms of adaptive immune responses. If the process described above occurs in antigen-presenting cells (macrophages or dendritic cells), microorganisms can be processed within the vesicles and generate peptides to be presented in the class II context, thereby initiating a strong antibody response. Then, upon vesicle rupture, microorganism components can be released into the cytoplasm, favoring their presentation in the context of class I molecules and generating cytotoxic T cell responses [114,115]. Thus, the presence of MPs may actually broaden the breadth of specific adaptive immunity against microorganisms by promoting both class II- and class I-dependent specific immunity. Based on this perspective, new vaccine adjuvants/carriers are being designed that include phago-lysosomal membrane destabilization as a preferred feature.

## Figures and Tables

**Table 1:** Interaction between human immunity and microorganisms carried by microplastics.

**Microplastic origin**	seawater, freshwater, soil, airborne dusts, cosmetics, textiles, food packaging
**Exposure route**	dermal, inhalation, ingestion
**Microorganisms on MPs**	viruses, bacteria; selection/concentration of specific strains, biofilm formation
**Main immune cells involved**	macrophages, neutrophils, mast cells (innate immune cells)
**Main soluble immune factors involved**	antimicrobial peptides, complement components (innate immune factors)
**Effects**	changes in the modality of infection (facilitation of entry via phagocytosis)
**Detrimental**	increased entry into target cells
**Beneficial**	increased microorganism intracellular killing, antigen processing and presentation, and establishment of adaptive immunity

## Data Availability

Not applicable.
